# Potential Markers to Differentiate Uterine Leiomyosarcomas from Leiomyomas

**DOI:** 10.7150/ijms.93464

**Published:** 2024-05-13

**Authors:** Jialu Guo, Jianfeng Zheng, Jinyi Tong

**Affiliations:** 1 Department of Obstetrics and Gynecology, Affiliated Hangzhou First People's Hospital, Westlake University School of Medicine, 310003 Hangzhou, Zhejiang Province, China.; 2 Department of Gynecology, Clinical Oncology School of Fujian Medical University, Fujian Cancer Hospital, 350014 Fuzhou, Fujian Province, China.

**Keywords:** uterine leiomyoma, leiomyosarcoma, sarcoma, marker, diagnosis

## Abstract

Uterine leiomyomas (ULM) are the most common benign tumors of the female genitalia, while uterine leiomyosarcomas (ULMS) are rare. The sarcoma is diffuse growth, prone to hematogenous metastasis, and has a poor prognosis. Due to their similar clinical symptoms and morphological features, it is sometimes difficult to distinguish them, and the final diagnosis depends on histological diagnosis. Misdiagnosis of ULM as ULMS will lead to more invasive and extensive surgery when it is not needed, while misdiagnosis of ULMS as ULM may lead to delayed treatment and poor prognosis. This review searched and studied the published articles on ULM and ULMS, and summarized the potential markers for the differential diagnosis of ULMS. These markers will facilitate differential diagnosis and personalized treatment, providing timely diagnosis and potentially better prognosis for patients.

## Introduction

Uterine tumors can be divided into benign and malignant. The most common benign uterine tumors are uterine leiomyomas (ULM), and the most common malignant uterine tumors are endometrial carcinoma and uterine sarcoma. Fibroids or myomas, often known as uterine leiomyomas, are the most prevalent benign tumors of the female genitalia, which occurs in 1 out of every 4 to 5 women 1, 2. Despite its benign nature, it has a high incidence. Because fibroids are often asymptomatic or rarely symptomatic, the reported incidence is much lower than the true incidence of fibroids 3. Common symptoms of uterine leiomyomas include increased menstrual bleeding, anemia, lower abdominal mass, infertility, etc., so it also troubles most women with uterine leiomyomas 4. Uterine leiomyosarcomas (ULMS) have similar clinical symptoms, but it is less common in comparison. They are the most common sarcoma of the uterine body. Three to nine percent of uterine malignancies are uterine sarcomas. ULMS have an extremely poor prognosis, are prone to hematogenous metastasis, and make up 60%-70% of all uterine sarcomas 5. According to a study, 42% of individuals with uterine leiomyosarcomas survive for the whole five years 6.

Although uterine leiomyomas are non-malignant, studies have found that one in 498 uterine tumors has a hidden risk of undiagnosed malignant tumors, such as leiomyosarcomas 7, 8. Uterine leiomyomas and uterine leiomyosarcomas have similar clinical symptoms and morphological features 9, and it is sometimes difficult to distinguish them, and the diagnosis is based on histological examination 10, 11. Surgery is considered the main treatment for ULM and ULMS 12, 13, so preoperative diagnosis is very important. If the preoperative diagnosis of ULMS is misdiagnosed as ULM, using morcellators during the operation will bring the risk of sarcoma spread 14, 15. As a result of a safety communication issued by the US Food and Drug Administration (FDA) in 2014, minimally invasive surgery in women with ULM is now severely limited due to the inability to use morcellators 16. Preoperative diagnosis of ULMS is challenging, if the ULM is misdiagnosed as ULMS, it will lead to more invasive and extensive surgery when it is not needed, while misdiagnosis of ULMS as ULM may lead to delayed treatment and poor prognosis 17.

Importantly, diagnostic methods such as ultrasound, CT, MRI, and CA-125 detection alone cannot accurately distinguish between malignant and benign uterine fibroids. Although CA125 is frequently utilized in daily practice, it is typically only markedly increased in advanced disease. And only 35 percent of cases could be diagnosed with ULMS by endometrial biopsy, according to Sagae *et al.* 18. At present, markers that can differentiate between uterine fibroids and sarcomas have not been found 19. Therefore, this study aims to find and summarize the potential diagnostic markers between ULM and ULMS by searching and studying the articles related to ULM and ULMS published so far. These potential diagnostic markers will facilitate the identification of ULM and ULMS, which will help to identify new therapeutic targets, which will not only effectively distinguish ULM from ULMS, but also accurately treat and even monitor prognosis.

## Materials and Methods

The source that we used was the PubMed database. Uterine leiomyoma, leiomyosarcoma, marker, biomarker, and diagnostic were the search phrases that were employed. 421 abstracts were assessed by title from 1122 search results that were retrieved between 1983 and 2024, 249 articles were extensively read, and 117 papers were ultimately included. Studies were not restricted by design, publication date, or number of patients reported due to the rarity of uterine leiomyosarcomas.

## Potential Markers

**Figure 1:**
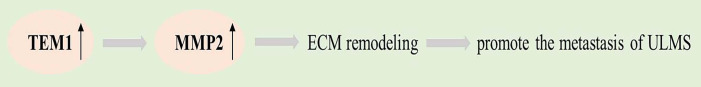
Graphical representation of the pathways involved in TEM1.

**Figure 2:**
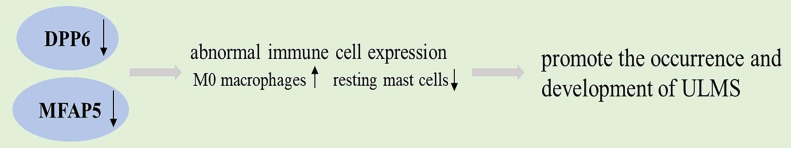
Graphical representation of the pathways involved in DPP6 and MFAP5.

**Figure 3:**
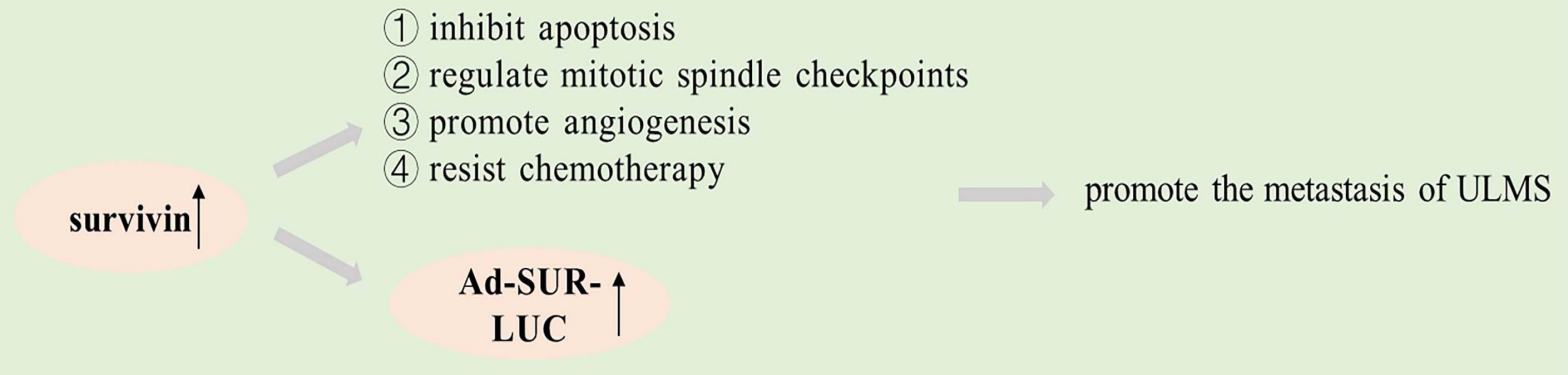
Graphical representation of the pathways involved in survivin.

**Figure 4:**
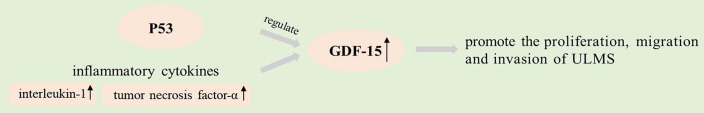
Graphical representation of the pathways involved in GDF-15.

**Figure 5:**
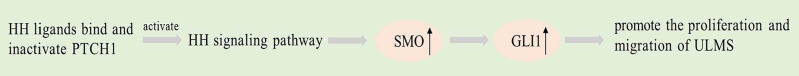
Graphical representation of the pathways involved in SMO.

**Figure 6:**
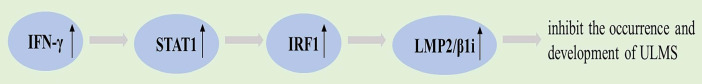
Graphical representation of the pathways involved in LMP2/β1i.

We searched the articles, summarized and described 16 potential diagnostic markers. Table 1 presents a summary of the markers. Some of the markers involved in some pathways we have added relevant graphical representation (Figures 1-6).

### Tumor Endothelial Marker 1 (TEM1)

TEM1 (also known as CD248 or endosialin) is a cell membrane protein that is mainly expressed in malignant tissues or during embryonic development, but hardly expressed in benign and normal tissues 20, 21. Its expression has been detected in skin cancer, colorectal, breast and other malignant tumors 22-24. It has been found that TEM1 is functional in controlling the interactions between tumor cells, endothelium, and stroma, and that TEM1 expression in tumor stroma and vascular endothelial cells may support tumor progression and invasion 24, 25. In addition, TEM1 is highly expressed in sarcomas. One study found that TEM1 was expressed in 96% of human sarcoma tissues among 19 sarcoma subtypes 26. TEM1 is an important therapeutic target for human sarcoma. Therefore, TEM1 may influence the onset and progression of uterine leiomyosarcomas and is anticipated to be a possible target for therapy.

Wu *et al.* found that TEM1 promotes the invasion and migration of ULMS by promoting extracellular matrix (ECM) remodeling through up-regulation of MMP-2 27. MMP-2 and TEM1 are co-expressed and positively correlated in ULMS whereas they were not expressed in ULM. It was found that MMP-2 activity and expression were upregulated in TEM1-overexpressing cells, whereas the opposite was observed in TEM1-knockdown cells. Depletion of MMP-2 suppressed the invasion and migration of TEM1-overexpressing cells. MMP-2 has been shown to be associated with a number of carcinomas metastasis 28, 29. For malignant tumors, cell-ECM adhesion is the key to their distant metastasis 30, 31. Wu *et al.* suggested that TEM1 can promote the adhesion of sarcoma cells to ECM components, and TEM1 overexpression also promotes sarcoma metastasis 27. This indicates that for the diagnosis of benign and malignant uterine fibroids, TEM1 is probably a good marker.

### Dipeptidyl Peptidase Like 6 (DPP6) and Microfibril Associated Protein 5 (MFAP5)

DPP6, a type II transmembrane protein derived from the ubiquitous family of serine peptidases in both prokaryotes and eukaryotes, is essential for the normal function of cells 32. Furthermore, MFAP5, an extracellular matrix (ECM) glycoprotein, is implicated in cell survival, elastinogenesis, and signaling during microfibril construction 33. Ke *et al.* found that DPP6 and MFAP5 were expressed at significantly lower levels in ULMS than in ULM, and the area under the ROC curve (AUC) determined that DPP6 and MFAP5 had the diagnostic ability to distinguish ULMS from ULM (AUC values were 0.957 and 0.899, respectively) 34. Studies have shown that the immune cell components of ULM and ULMS are different, and DPP6 and MFAP5 are also associated to infiltrating immune cells 34, 35. DPP6 was positively correlated with some immune cells that were more abundant in ULM and negatively correlated with some immune cells that were more abundant in ULMS. For example, the proportion of macrophage M0 was notably higher in uterine leiomyosarcomas than in leiomyomas, and DPP6 was negatively related to M0 macrophages. Additionally, MFAP5 was positively related to resting mast cells, and the ratio of resting mast cells was significantly higher in ULM than in ULMS 34. Therefore, the association of DPP6 and MFAP5 with immune cells can speculate that they may have an impact on immune-related pathways that influence the development and incidence of ULMS. In malignant tumors, more and more studies use immune cells as a novel research direction for diagnosing diseases and prognosis 36-38. For example, in patients with breast cancer, low DPP6 expression predicts unfavorable prognosis, which is consistent with Ke *et al.* 's finding that DPP6 expression level in ULMS is significantly lower than that in ULM 39.

KEGG analysis revealed that immune-related and cell cycle-related pathways, including the HTLV-1 infection pathway, were enriched in the differentially expressed genes between ULM and ULMS 34. Related studies have shown that HTLV-1 enhances genomic instability through changing host genes expression directly, thereby affecting immune-related pathways and leading to malignant transformation 40, which again demonstrates that the regulation of immune response may be closely related to the occurrence of ULMS.

### Tumor Protein p53, p16 and Ki67

P53, p16 and Ki67 have been studied more in immunohistochemistry and are often used to distinguish benign and malignant lesions 41, 42. In human cancers, mutations in the p53 tumor suppressor gene are frequently found 43. The most prevalent gene mutation in solid tumors, p53 mutations, can make cells resistant to the activation of intrinsic apoptotic pathways 44. De Vos *et al.* first proposed that p53 gene mutations are more common in ULMS. They claim that the acquisition of p53 mutations is a difference between leiomyomas and leiomyosarcomas 45. Nordal *et al.* suggested that p53 gene abnormalities possibly play an essential part in the development of uterine sarcoma 46. Previous researches have shown that the expression of p53 between ULMS and ULM is significantly different 47, 48. The immunohistochemical staining pattern of p53 mutation is significantly higher in ULMS than in ULM 49.

The overexpression of p16 is also described in ULMS. P16 is a tumor inhibitory protein that plays a vital role in regulating the cell cycle. It acts as a negative regulator of the cell cycle by binding to the cell cycle-dependent kinase CDK4-cyclin D^47^, ^50^, ^51^. P16 normally promotes growth arrest, but increased expression in tumor cells causes protein accumulation in their nuclei and cytoplasms, and strong and diffuse p16 positivity is observed in immunohistochemistry 50. In uterine leiomyosarcomas, p16 gene expression appears to be upregulated, and this upregulation may also extend to p16 protein expression, which has been reported by several studies in ULMS compared with ULM 47, 52, 53. In uterine leiomyomas, aberrant expression of p53, p16 has been described as a hallmark of malignancy 47.

Abnormal cell proliferation is the main reason for the occurrence and development of carcinogenic processes. Ki67(also known as MKI67) is a proliferation marker and a nuclear DNA binding protein 54. Ki-67 is a late marker of cell cycle entry in normal cells and is strongly downregulated in quiescent G0 phase cells, with the highest expression of Ki-67 mRNA in G2 phase, whereas Ki-67 protein expression increases throughout the cell cycle and peaks in mitosis 54, 55. Ki-67 is highly expressed in endometrial cancer 56, ovarian cancer 57, and cervical cancer 58, and high Ki-67 index generally indicates poor clinical prognosis 59. Compared to uterine leiomyomas, uterine leiomyosarcomas express significantly higher Ki67 mRNA levels 60. The increased expression of Ki67 is also considered as a diagnostic marker for the malignancy of uterine leiomyosarcoma 61, and high Ki67 is associated with poor prognosis of leiomyosarcoma 41, 42.

### Survivin

The survivin gene encodes an inhibitor of apoptosis (IAP) that is structurally unique to humans. Studies have shown that survivin is significantly expressed in many human cancers, such as lung carcinoma, breast carcinoma and colon carcinoma 62-64. The survivin protein plays a key role in mitosis and programmed cell death, and a genome-wide search indicated that survivin expression differs in normal and tumor tissue 65. It may be a universal characteristic of tumorigenesis that apoptosis is inhibited, thereby preventing normal homeostasis and promoting tissue tumorigenesis 66, and survivin inhibits apoptosis, regulates mitotic spindle checkpoints, promotes angiogenesis, and resists chemotherapy in cancer pathogenesis, according to studies 44, 67. Increased expression of survivin is an adverse prognostic marker in patients, and a high expression of survivin is also linked to an increase in recurrences, lymphadenopathy, and metastasis 68, 69. Survivin has been identified as a cancer-specific promoter in lots of researches 70.

Shalaby *et al.* found that survivin expressed its downstream reporter gene in ULMS merely but not in fibroids. Compared with uterine leiomyoma, the downstream reporter gene (Ad-SUR-LUC) of survivin promoter was highly expressed in uterine leiomyosarcoma cells 71. Their study showed that survivin is a promoter that can distinguish ULMS from ULM. It will be possible to detect cancer cells by using the expression of survivin in different cells as a method for testing the promoter driving power of the downstream reporter gene, which can serve for the early detection of cancer cells 72. Intravenous injection of Ad-SUR-LUC in the study by Shalaby *et al.* successfully differentiated preexisting human leiomyosarcoma from human uterine leiomyoma in a mouse model 71. Therefore, survivin may be a promising new target for cancer therapies that are based on apoptosis.

### Growth Differentiation Factor-15 (GDF-15)

GDF-15, also known as macrophage inhibitory cytokine-1 (MIC-1), is a secreted cytokine regulated by P53, which is associated with tumorigenesis and is a biomarker for ovarian and endometrial cancer 73-75. Inflammation is considered one of the "hallmarks of cancer" and interleukin-1 and tumor necrosis factor-alpha may activate macrophages and induce GDF-15 production 76, 77. Trovik *et al.* showed that uterine leiomyosarcoma patients had significantly higher circulating GDF-15 levels compared to leiomyomas patients, and the ROC curve analysis showed that GDF-15 has a certain accuracy in the diagnosis of ULMS and ULM, indicating that GDF-15 is an effective biomarker to distinguish ULM and ULMS 78. The high expression of GDF-15 in ULMS may be due to the activation of cancer-related inflammatory processes.

GDF-15 has a certain correlation with p53 and macrophages, so it is speculated that the high expression of GDF-15 in ULMS may be due to the activation of cancer-related inflammatory process 77, or it may be the target of downstream pathways regulating cell cycle arrest and apoptosis, and has an impact on cancer proliferation, migration, invasion, etc. 79.

### Minichromosome Maintenance Complex Component 2 (MCM2)

MCM2 belongs to the family of MCM proteins. It plays a key role in DNA replication initiation and replication fork movement and is closely associated with cell proliferation, with its protein levels increasing in G1 and peaking in S1^80^, ^81^. MCM2 overexpression has been found in a wide variety of malignant tumors in recent years, and as a promising proliferation marker, it is expected to become a marker for the identification of malignant tumors 80, 82, 83.

According to Quade *et al.*, MCM2 expression was 18.2-fold higher in ULMS than in ULM, that is, MCM2 expression increased significantly in ULMS compared to ULM 84. The population studied by Keyhanian *et al.*, ULMS patients consistently demonstrated a high proportion of MCM2 responses, the sensitivity and specificity for diagnosing ULMS with MCM2>80% were 92% and 94%, respectively 85. This study suggests that MCM2 is valuable in identifying ULMS. and additionally, combining MCM2 with p16 or Ki67 can better distinguish ULMS from ULM 85.

### Stathmin 1 (STMN1)

STMN1, also known as oncoprotein 18, is a microtubule depolymerization-related protein that is widely expressed in the cytoplasm. It exerts regulatory control over microtubule dynamics through the inhibition of tubulin polymerization and the promotion of microtubule instability, while also facilitating tumor cell proliferation, differentiation, and invasion 86-88. STMN1 is expressed in various carcinomatosis such as ovarian cancer 89, endometrial cancer 90, cervical cancer 91, hepatocellular carcinoma 92, and bladder cancer 93, and inhibition of STMN1 can reduce cell viability and migration potential.

The activation of the oncogenic phosphatidylinositol 3-kinase-AKT-mammalian target of rapamycin pathway (PI3K-AKT-mTOR) has been observed in uterine smooth muscle tumors. Furthermore, the identification of STMN1 expression serves as an indicator for the activation of the PI3K-AKT-mTOR pathway 94. Hwang *et al.* showed that the expression of STMN1 has some value in differentiating uterine fibroids from uterine sarcomas 95. The research by Allen *et al.* found that STMN1 expression in uterine leiomyosarcomas was mainly diffusely and strongly positive, while uterine leiomyomas was mainly weakly positive. The expression of STMN1 exhibited a sensitivity of 100% in relation to leiomyosarcomas, yet its specificity was merely 55% 94. Consequently, STMN1 emerges as a remarkably sensitive indicator for leiomyosarcomas, albeit with limited specificity for diagnostic applications. In line with the findings of Allen *et al.*, Hu *et al.* also discovered that STMN1 expression in uterine leiomyosarcomas was much higher than that in uterine leiomyomas 60, 94. Therefore, STMN1 is a gene associated with tumors and is a possible target for diagnosis and treatment.

### Smoothened (SMO) and GLI Family Zinc Finger 1 (GLI1)

Dysregulation of the Hedgehog (HH) pathway has been documented in ULMS patients, with higher expression levels of GLI1 and SMO in ULMS compared to ULM. The evidence presented suggests a correlation between HH pathway dysregulation and ULMS development 96, 97. The HH pathway signaling involves three ligands: sonic hedgehog (SHH), Indian hedgehog (IHH), and desert hedgehog (DHH), two receptors: PTCH1 and SMO, and three transcription factors: GLI1, GLI2 and GLI3 mediate. When HH ligands bind and inactivate PTCH1, the classical HH signaling pathway is activated, releasing SMO protein signals to its cytoplasmic targeting 98. SMO triggers the translocation of GLI proteins to the nucleus, which leads to their subsequent binding to DNA 99. GLI members are nuclear regulators situated at the pathway's end and in charge of controlling the downstream target genes' expression 100.

The HH signaling pathway has been implicated in tumorigenesis and cell differentiation in several studies 96, 101. Analyzing the protein expression in components of Hedgehog signalling in uterine smooth muscle tumors is helpful for the diagnosis, prognosis or malignant risk prediction of uterine smooth muscle tumors 96. Garcia *et al.* found activation of the Hedgehog pathway and increased GLI nuclear translocation in ULMS. SMO and GLI1 expression in ULMS is higher than that in ULM 97. PTCH1 expression is downregulated in ULMS. SMO or GLI inhibitors applied to ULMS cells inhibit cell proliferation and migration while inducing apoptosis 97. In the future, GLI1 and SMO may become therapeutic targets for uterine smooth muscle tumors.

### Lethal-7 (let-7) miRNA, miR-191-5p and miR-1246

MicroRNAs (miRNAs) regulate the expression of a variety of target genes in cells, mainly through regulating the translation of the target genes 102. Studies have shown that extracellular miRNAs have significant functions in cell-to-cell communication and various biological mechanisms 103. Altered expression of miRNAs may lead to tumorigenesis, and gynecologic tumors such as ovarian cancer, endometrial cancer, cervical cancer, and uterine sarcoma are associated with unregulated expression of miRNAs 104, 105. Blood contains miRNAs at a stable concentration, and it has been established that circulating miRNAs can be used as disease biomarkers 106.

The let-7 family of miRNAs is a key regulator of eukaryotic cell apoptosis, differentiation and pluripotency. The let-7 family is a major family of miRNAs that normally function as tumor suppressors 107. The study's findings demonstrated that all members of the let-7 family had downregulation in ULMS, and that a worse patient prognosis was correlated with a higher degree of loss of expression 108. This suggests that let-7 is a potential prognostic biomarker for LMS. Loss of let-7 likely result from perturbation of the signalling network involving key families of proteins, leading to an acceleration of tumor progression 109. Downregulation of let-7 is prevalent in lots of cancers, and substitution of let-7 for normal expression has been shown to arrest tumor growth 110.

Furthermore, Yokoi *et al.* endeavored to ascertain diagnostic biomarkers that could effectively differentiate between ULMS and ULM through their investigation of circulating miRNAs. Their primary objective was to identify potential miRNAs that could be utilized in the development of a diagnostic model for ULMS, and seven candidate miRNAs (miR-191-5p, miR-1246, miR-4635, miR-4485-5p, miR-451a, miR-6511b-5p and miR-4430) were screened out 111. These seven miRNAs were significantly downregulated in ULMS. The optimal model consisted of two miRNAs (miR-191-5p and miR-1246) based on the model construction, and ULMS patients could be accurately identified using this dual miRNA prediction signature 111. Therefore, the combination of miR-191-5p and miR-1246 is a potential marker for differentiating ULMS from ULM.

### Major Vault Protein (MVP)

MVP, also called lung resistance-related protein (LRP), is located on chromosome 16 and helps move various molecules in and out of signal transduction networks and toxic compounds out. Data indicate high MVP expression is related with resistance to multiple chemotherapy regimens 112. In acute myeloid leukaemia, lung cancer, ovarian cancer and other malignant tumors, its increased expression has been found to be associated with the induction of multidrug resistance 112, 113. By analyzing differentially expressed proteins between ULMS and ULM, Lintel *et al.* shown that MVP expression in ULMS was 3.05-fold higher than that in ULM. By immunohistochemistry (IHC), MVP found 50% sensitivity and 100% specificity when comparing ULMS and ULM 114. MVP is a helpful adjunct for differentiating ULMS from ULM, although negative staining results cannot rule out malignancy, positive staining results are a strong indicator of malignancy 114.

### Large Multifunctional Protease 2 (LMP2/β1i)

LMP2/β1i is an immunoproteasome catalytic subunit 115. Mice with LMP2/β1i gene deficiency were found to spontaneously develop ULMS through animal models 116, 117. LMP2/β1i is not expressed in ULMS but is present in ULM. Thus, one of the risk factors for ULMS may be defective LMP2/β1i expression 117, 118. Tumor rejection mediated by MHC class I molecules, a process influenced by the function of the proteasome induced by interferon-γ (IFN-γ) 119, 120. The findings confirm that IFN-γ prevents the development of primary tumors and thus exhibits a tumor suppressive effect in the immune response 119, 121.

The expression of LMP2 is significantly induced by IFN-γ, and the activation of signal transducer and activator of transcription (STAT) 1 by IFN-γ leads to the upregulation of tumor suppressors, including interferon regulatory factor 1 (IRF1). IRF1 functions as a transcriptional regulator that plays a significant role in the regulation of LMP2 expression 122, 123. Decreased IRF1 level caused by LMP2 deficiency may be a risk factor for ULMS 118.

LMP2 immunostaining is helpful in the differential diagnosis of ULMS and ULM, as LMP2 protein expression is attenuated in 85% of ULMS samples 124. LMP2/β1i is a promising diagnostic marker for ULMS and has the potential to become a targeted molecule for novel therapies 117.

### Extracellular Matrix Metalloproteinase Inducer (EMMPRIN)

EMMPRIN, also known as CD147, is a member of the human immunoglobulin superfamily and an inducer of extracellular matrix proteolytic enzymes encoded by the BSG gene 125. EMMPRIN, which is abundantly expressed on the surface of tumor cells, assumes a pivotal function in the advancement of numerous cancers through its stimulation of matrix metalloproteinases (MMPs) and cytokine secretion 126, 127. MMPs are crucial in the degradation of the extracellular matrix and the progression of tumors 128, 129. The degradation of the extracellular matrix is of utmost significance in facilitating tumor invasion, growth, and metastasis. Consequently, EMMPRIN assumes a crucial role in cancer cells by governing cell proliferation, apoptosis, migration, metastasis, and differentiation, particularly in hypoxic environments 125. Many tumors, particularly disseminated cancer cells and those with a poor prognosis, express EMMPRIN at high levels 130-132. Studies have shown that EMMPRIN may serve as a potential early disease diagnostic marker. For example, EMMPRIN is considered a promising therapeutic target for the treatment of hepatocellular carcinoma, and monoclonal antibodies targeting EMMPRIN have made exciting clinical progress in the treatment of hepatocellular carcinoma 125.

In the study by Kefeli *et al.*, the degree and intensity of EMMPRIN staining and their combined score were compared between ULMS and benign uterine smooth muscle tumors, and the findings suggested that the difference was statistically significant, and the EMMPRIN expression in the LMS group was notably higher than in the ULM group 133. The results of Ozler *et al.* are consistent with those of Kefeli *et al.*, high EMMPRIN expression is predominantly observed in ULMS 134, indicating that EMMPRIN may serve as a useful immunohistochemical marker to distinguish LMS from other benign smooth muscle tumors.

### Promyelocytic Leukemia Zinc Finger (PLZF) and Histone H1.5

PLZF protein is a DNA-binding transcriptional repressor that negatively regulates the progression of the cell cycle, ultimately leading to growth inhibition 135. PLZF was found to be highly expressed in the secretory endometrium and the myometrium by IHC 136. However, its expression has been observed to decrease in lung cancer, melanoma and hematological malignancies 137-139. Histone H1.5 is a type of histone H1, which is a group of proteins that assist in organizing chromosomes into more complex structures 139-141. H1.5 has been found to have an effect on the regulation of transcriptional, and this protein is strongly expressed in lung neuroendocrine tumors and prostate cancer and is associated with disease progression 139, 142.

PLZF was under-expressed in ULMS, whereas H1.5 was over-expressed in ULMS. In uterine leiomyomas, the expression of the two proteins is opposite 143. Thus, as immunohistochemical biomarkers for ULMS and ULM, PLZF exhibits an inverse relationship with H1.5. This indicates that PLZF or H1.5 staining may serve as a useful screening test. The study by Momeni *et al.* showed that combining these two immunophenotypes resulted in a specificity and sensitivity of 97.5% and 90.5%, respectively, in differentiating ULM from ULMS 143.

### Insulin-like Growth Factor Ⅱ mRNA Binding Protein 3 (IMP3)

IMP3 is a member of insulin-like growth factor RNA binding protein family. IMP3, an oncogenic fetal protein linked to advanced and aggressive cancers, is expressed only in malignant tumors and is not found in benign tissues. It has a role in embryogenesis and carcinogenesis of certain malignant tumors 144-146. Research has demonstrated that IMP3 can stimulate the growth, invasion, and metastasis of tumor cells 146, 147. The study by Cornejo *et al.* suggested that IMP3 is strongly expressed in ULMS but not in benign leiomyomas 148. One extremely specific biomarker of leiomyosarcoma is IMP3, which may be involved in the pathophysiology of the disease. IMP3 is a cancer-specific biomarker linked to more aggressive tumor behavior, as demonstrated by earlier research on endometrial and renal cell carcinomas 144, 146, 149. IMP3 immunoreactivity in leiomyosarcoma also illustrates the more aggressive behavior of the tumor and predicts a worse prognosis. Therefore, to distinguish between benign and malignant smooth muscle tumors, IMP3 staining can be a helpful adjunct. Additionally, IMP3 expression in the uterus can be utilized as a positive biomarker to raise the degree of confidence in the final diagnosis of malignant smooth muscle tumors.

### Hepatocyte Growth Factor Activator Inhibitors (HAI)-1 and -2

HAI-1 and HAI-2 were originally described as endogenous inhibitors of hepatocyte growth factor activator (HGFA), matriptase, hepsin and prostasin. These proteolytic enzymes are primarily membrane-bound and may perform crucial functions in cellular homeostasis. Dysregulation of their activity and expression has been linked to the development and advancement of tumors 150-152. In the study by Nakamura *et al.*, HAI-1 and HAI-2 mediated cell invasion, migration and proliferation, leading to necrosis and apoptosis by reducing the expression of HGFA, hepsin and matriptase 153.

Hepatocyte growth factor (HGF) is a multifunctional growth factor that is secreted by liver mesenchymal cells and acts on the motility and morphogenesis of a variety of target cells, usually associated with the ECM 154. Degradation of the ECM facilitates cell separation, leading to local and systemic dissemination. HAI-1 and HAI-2 regulate HGFA, which is in charge of the proteolytic activation of the precursor forms of HGF in a variety of human cancer tissues and serum 155, 156. Reduced expression of HAI-1 and HAI-2 has been linked in the past to the advancement of ovarian and cervical cancer, according to research 157, 158. A range of serine proteases that may be implicated in carcinogenesis, invasion, and metastasis may be efficiently inhibited by HAI-1 and HAI-2, and their overexpression decreases cell adherence and spreading 153.

The typical human uterus has high levels of expression of both HAI-1 and HAI-2 in its surface epithelium and uterine glands 159. HAI-1 and HAI-2 expression levels in ULMS tissues were notably lower than those in ULM tissues 153. Furthermore, compared to high expression of HAI-1 and HAI-2, reduced expression of these genes was a strong indicator of a poor prognosis 153. Given the suggestion that HAI-1 and HAI-2 may be significant tumor suppressor genes for the detection of ULMS, both may be taken into consideration as therapeutic options for the illness.

### CD44 variant 3 (CD44v3)

As a transmembrane glycoprotein, CD44 is involved in intercellular and cell-matrix interactions 160. The simplest CD44 standard (CD44s) does not contain any additional exon products, whereas CD44 variants (CD44v) contain one or more additional exons. Studies have shown that alterations in CD44 protein are associated with tumorigenesis, local invasion, metastasis, recurrence, and poor prognosis 161-163.

CD44v3 was expressed in ULM but not in ULMS. When using CD44v3 negative staining to diagnose ULMS, the specificity, sensitivity, positive predictive value, and negative predictive value were all 100% 164. The expression of CD44s is decreased in ULMS relapse patients. The study by Poncelet *et al.* suggested that CD44s immunostaining in leiomyosarcomas may be prognostic and that loss of CD44v3 expression may serve as a potential diagnostic tool for uterine leiomyosarcomas 164.

## Other Biomarkers

In addition to some of the markers described above, there are other markers that are helpful in differentiating ULMS from ULM, which are briefly described as follows.

The expression of B-cell lymphoma 2 (Bcl2) and DNA fragmentation factors 40, 45 (DFF40, 45) was significantly lower in ULMS compared to ULM 165, 166, while progesterone and estrogen receptors (PR and ER) were either faintly positive or negative 167, 168. DFF40 and DFF45 are the ultimate DNA ladder leading to apoptosis, and Bcl2 is an inhibitor of apoptosis. Bcl2 expression changes in breast cancer, endometrial cancer, ULMS and other malignant tumors 169, 170. Low or absent expression of these markers is associated with potentially adverse outcomes 166, 171.

Mediator complex subunit 12 (MED12) mutations are the first recurrent oncogenic mechanisms identified in smooth muscle tumors 172. These somatic mutations have demonstrated reliability as biomarkers for uterine fibroids, with MED12 mutations being present in roughly 70% of affected women 173. MED12 mutations are rarely seen in ULMS 174, 175. The alteration of MED12 may be related to the occurrence of smooth muscle tumors, and its expression may be inhibited in malignant tumors 176. Furthermore, the Wilms' tumor 1 (WT1) that is expressed in various gynecologic tumors, such as endometrial stromal tumors and ovarian cancer 177, 178. It has been found that uterine leiomyosarcomas is more likely to have a loss of WT1 expression, and WT1 is expressed in most uterine leiomyomas, so WT1 may also have a certain value in distinguishing benign and malignant uterine leiomyosarcomas 49, 178.

Besides, immunohistochemistry showed that the expression of hyperthermia inhibited histone acetyltransferase 1 (HAT1) in ULMS was higher than that in ULM, and was associated with poor prognosis. Studies suggest that further preclinical investigation of HAT1 as a promising drug target for treating ULMS is warranted, particularly when combined with hyperthermia 179. And there was a notable contrast in lactate dehydrogenase (LDH) levels observed among patients with benign uterine masses and sarcomas. Serum LDH levels may be elevated in patients with ULMS, but the sensitivity is low, and it is difficult to identify ULMS by LDH detection alone 180, 181. Di Cello *et al.* found that LDH3, LDH4, and LDH5 isoenzymes were notably higher in uterine sarcoma patients compared to those with uterine fibroids, while LDH1 and LDH2 were significantly lower 182. It has been found that in addition to LDH, D-dimer may be elevated in ULMS and C-reactive protein is highly positive, and the combined detection of these three is useful in the differential diagnosis of ULMS and ULM 183.

Studies have demonstrated the involvement of carbonic anhydrases (CAs) in relation to different types of cancer, whereby they aid in regulating pH homeostasis in the microenvironment of tumor cells 184. CA isoenzymes can be used as a histopathological biomarker for the differential diagnosis of ULMS and ULM. CA isoenzymes are absent in most fibroids, whereas all uterine leiomyosarcomas show positive staining 185.

## Discussion

ULMS is a disease that exhibits a high mortality rate, a high recurrence rate, and the prognosis is poor. The pathogenesis of ULMS is still unclear, and there is no specific biomarker that can be used for differentiating it from other analogues. Consequently, the preoperative detection of ULM and ULMS presents significant challenges. ULM are diagnosed by means of pelvic examination, ultrasonography, and, if necessary, enhanced magnetic resonance imaging (MRI), but it is not straightforward to use contrast-enhanced magnetic resonance imaging or other clinical tests to determine the degree of malignancy of uterine smooth muscle tissue or to diagnose a mass 9. If the above examination is not very definite as ULM, or if the patient's mass is rapidly growing or is postmenopausal, then the possibility of a malignant uterine tumor such as ULMS should also be considered 186. Clinically, ULMS may be found more incidentally by performing histopathological examination of specimens 10. Therefore, ULMS and ULM cannot be reliably differentiated due to the absence of specific symptoms or diagnostic imaging studies. Currently, surgery remains the sole viable approach for diagnosis and treatment. Myomectomy is usually performed with minimally invasive procedures such as laparoscopic surgery 10, 11. Minimally invasive surgery for a presumed benign uterine leiomyoma may result in unexpected intra-abdominal spread of sarcoma, resulting in poor survival 13, 187. For the safety of the patient with regard to tumors, alternative invasive methods, including procedures based on open surgery, lead to higher morbidity, mortality, and costs for both the patient and the healthcare system 187. Given these challenges, it is crucial to prioritize the development of highly accurate biomarkers and non-invasive diagnostic methods in fields like gynecology and oncology. The purpose of this review is to analyze the available literature on the genes or proteins that differ between ULM and ULMS to provide valuable insights for the differential diagnosis of ULM and ULMS. It aims to aid in identifying specific diagnostic markers and therapeutic targets for ULMS and ULM, with the potential to establish a foundational basis for future assessment of malignant risk.

The search for a reliable and easily accessible method to distinguish ULMS from ULM has been the subject of much research. By summarizing the previous literature, we found many candidate molecules for the differential diagnosis between ULMS and ULM, and we selected some of them to describe. Compared with ULM, TEM1, MMP-2, p53, p16, Ki67, survivin, GDF-15, MCM2, STMN1, SMO, GLI1, MVP, EMMPRIN, H1.5 and IMP3 were up-regulated in ULMS. DPP6, MFAP5, let-7 miRNA, miR-191-5p, miR-1246, LMP2, PLZF, HAI-1, HAI-2 and CD44V3 were down-regulated in ULMS. Table 1 shows an overview of the biomarkers. In total, 489 genes with differential expression between ULMS and ULM were identified, wherein 416 were notably upregulated while 73 were notably downregulated. These discrepancies primarily participate in the mechanism of genes related to the cell cycle 188. For instance, ULMS has been linked to numerous rearrangements that target the chromatin remodeling protein ATRX 188. ATRX expression loss is linked to a telomere phenotype that is selectively prolonged, enabling tumor cells to evade planned cell death. ULMS has a correlation between this mechanism and poor prognosis and overall survival 189, 190. In addition, the cyclin AURKA also appears to be critical for ULMS pathogenesis, as it inhibits cell-cycle arresting and apoptosis of ULMS cell lines 191. AURKA is also overexpressed in ovarian and cervical cancer 192, 193.

PD-L1 expression and cytotoxic T-cell infiltration were significantly higher in ULMS compared to ULM, suggesting a possible role for PD-1/PD-L1 checkpoint inhibition in the leiomyosarcoma patient population 194. The expression of TOP2A, which is significantly elevated in ULMS but not in nonmalignant smooth muscle diseases, may also be an important diagnostic tool for difficult cases in which the diagnosis of ULMS is not clear 195. The expression of cellular retinol binding protein-1 (CRBP-1) in ULMS is higher than that in ULM, and CRBP-1 overexpression is linked to alterations in signaling molecules for cell proliferation and apoptosis 196. It has also been suggested that methylation level can be used to distinguish ULMS from ULM. Brany *et al.* reported that methylation events in benign fibroids and sarcomas should have different patterns, and the KLF4 and DLEC1 genes can be considered as potential methylation biomarkers for uterine fibroids 197.

In recent years, more studies tend to combine multiple indicators to distinguish uterine leiomyoma and uterine leiomyosarcoma. The detection of LDH, D-dimer, and C-reactive protein in combination is beneficial for distinguishing between ULMS and benign lesions. Specificity and positive predictive value were found to be 100% when all three of these markers were combined. In addition, applying these three markers in combination with MRI will allow more accurate diagnosis of ULMS, and prospective studies of these markers and MRI results are warranted in the future 183. The combined detection of MCM2, Ki67 and p16 immunohistochemistry has good diagnostic value in the differential diagnosis of ULMS 85. Furthermore, the study of collagen in ULM may also help to distinguish ULM from ULMS. In ULM, there is excessive deposition of ECM, the major component being collagen, which is involved in keeping the tissue's structural integrity 198. Collagens have the ability to alone or in conjunction with integrins and growth factor-mediated mitogenic pathways to modify the behavior and function of cells 199. It has been proposed that aberrant collagen fiber orientation and structure are present in ULM, and that changes in collagen genes may contribute to the pathophysiology of leiomyomas 200. Thus, the microstructure collagen properties of ULM can be characterized using an inventive multidisciplinary method based on Phase-Contrast MicroComputed Tomography, Transmission Electron Microscopy, and Fourier Transform Infrared Imaging Spectroscopy 198.

The treatment and prognosis of ULM and ULMS are different. Accurate differentiation of ULM and ULMS is of great significance for formulating appropriate treatment plans, evaluating prognosis and improving patients' quality of life. While some studies can serve as a basis for future research on the pathophysiology and diagnosis of ULMS, the findings of studies on candidate molecules for the differential diagnosis of ULM and ULMS cannot yet be applied clinically. Based on the molecular properties of ULMS tissue, differentiated diagnosis and tailored treatment can result in an earlier diagnosis and a better prognosis.

## Author Contributions

JT designed the study. JG drafted the manuscript. JZ revised the manuscript. All authors have read and approved the final manuscript.

## Figures and Tables

**Table 1:** Potential biomarkers for differential diagnosis between ULMS and ULM.

Biomarker	Expression in ULMS	Reference
TEM1, MMP-2	Up	26, 27
DPP6, MFAP5	Down	34
p53, p16, Ki67	Up	45-49, 61
survivin	Up	71
GDF-15	Up	78
MCM2	Up	84, 85
STMN1	Up	60, 94
SMO, GLI1	Up	96, 97
let-7 miRNA	Down	108
miR-191-5p, miR-1246	Down	111
MVP	Up	114
LMP2/β1i	Down	118, 124
EMMPRIN	Up	133, 134
PLZF	Down	143
H1.5	Up	143
IMP3	Up	148
HAI-1, HAI-2	Down	153
CD44V3	Down	164

## Abbreviations

ULM: uterine leiomyomas
ULMS: uterine leiomyosarcomas
TEM1: tumor endothelial marker 1
ECM: extracellular matrix
DPP6: dipeptidyl peptidase like 6
MFAP5: microfibril associated protein 5
GDF-15: growth differentiation factor-15
MIC-1: macrophage inhibitory cytokine-1
MCM2: minichromosome maintenance complex component 2
STMN1: stathmin 1
HH: hedgehog
SMO: smoothened
GLI1: GLI family zinc finger 1
miRNAs: microRNAs
let-7: lethal-7
MVP: major vault protein
LRP: lung resistance-related protein
IHC: immunohistochemistry
LMP2/β1i: large multifunctional protease 2
IFN-γ: interferon-γ
STAT: signal transducer and activator of transcription
IRF1: interferon regulatory factor 1
EMMPRINE: extracellular matrix metalloproteinase Inducer
MMPs: matrix metalloproteinases
PLZF: promyelocytic leukemia zinc finger
IMP3: insulin-like growth factor Ⅱ mRNA binding protein 3
HAI: hepatocyte growth factor activator inhibitors
HGFA: hepatocyte growth factor activator
HGF: hepatocyte growth factor
CD44s: CD44 standard
CD44v: CD44 variants
DFF40: DNA fragmentation factors 40
Bcl2: B-cell lymphoma 2
MED12: mediator complex subunit 12
PR: progesterone receptor
ER: estrogen receptor
WT1: Wilms' tumor 1
HAT1: hyperthermia inhibited histone acetyltransferase 1
LDH: lactate dehydrogenase
CAs: carbonic anhydrases
MRI: magnetic resonance imaging
CRBP-1: cellular retinol binding protein-1
